# 4-(2,4-Dichloro­phen­yl)-6-(1*H*-indol-3-yl)-2,2′-bipyridine-5-carbonitrile

**DOI:** 10.1107/S1600536809012392

**Published:** 2009-04-08

**Authors:** P. Ramesh, S. S. Sundaresan, P. Thirumurugan, Paramasivan T. Perumal, M. N. Ponnuswamy

**Affiliations:** a Department of Physics, Presidency College (Autonomous), Chennai 600 005, India; b Centre of Advanced Study in Crystallography and Biophysics, University of Madras, Guindy Campus, Chennai 600 025, India; c Organic Chemistry Division, Central Leather Research Institute, Adyar, Chennai 600 020, India

## Abstract

The title compound, C_25_H_14_Cl_2_N_4_, crystallizes with two independent mol­ecules in the asymmetric unit. The two pyridine rings are almost coplanar, making dihedral angles of 3.2 (1) and 8.6 (1)° in the two independent mol­ecules. The dichloro­phenyl and indole rings are twisted away from the bipyridine ring by 64.32 (5) and 18.46 (4)°, respectively in the first molecule and by 51.0 (1) and 27.99 (5)°, respectively in the second molecule. The crystal packing is stabilized by C—H⋯N, C—H⋯Cl, N—H⋯N and C—H⋯π inter­actions.

## Related literature

For the use of pyridine derivatives containing cyano, amino, carboxyl and hydroxyl groups as drugs, see: Zhou *et al.* (2008 ▶); Stevenson *et al.* (2000 ▶); Harris & Uhle (1960 ▶); Ho *et al.* (1986 ▶); Rajeswaran *et al.* (1999 ▶). For hydrogen-bond motifs, see: Bernstein *et al.* (1995 ▶).
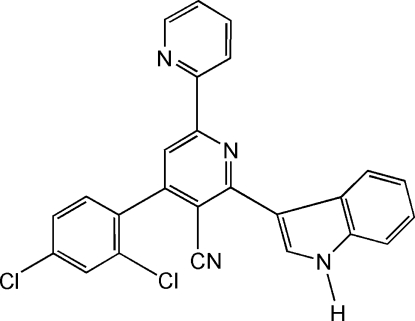


## Experimental

### 

#### Crystal data

C_25_H_14_Cl_2_N_4_
*M* *_r_* = 441.30
Monoclinic, 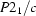

*a* = 10.0307 (12) Å
*b* = 22.446 (3) Å
*c* = 17.932 (3) Å
β = 90.991 (4)°
*V* = 4036.7 (10) Å^3^
*Z* = 8
Mo *K*α radiation
μ = 0.34 mm^−1^
*T* = 293 K
0.30 × 0.25 × 0.20 mm

#### Data collection

Bruker Kappa APEXII area-detector diffractometer
Absorption correction: multi-scan (*SADABS*; Sheldrick, 2001 ▶) *T*_min_ = 0.902, *T*_max_ = 0.934
38628 measured reflections
7874 independent reflections
5586 reflections with *I* > 2σ(*I*)
*R*_int_ = 0.038

#### Refinement

*R*[*F* ^2^ > 2σ(*F* ^2^)] = 0.043
*wR*(*F* ^2^) = 0.141
*S* = 1.06
7874 reflections
567 parameters
H atoms treated by a mixture of independent and constrained refinement
Δρ_max_ = 0.52 e Å^−3^
Δρ_min_ = −0.41 e Å^−3^

### 

Data collection: *APEX2* (Bruker, 2004 ▶); cell refinement: *SAINT* (Bruker, 2004 ▶); data reduction: *SAINT*; program(s) used to solve structure: *SHELXS97* (Sheldrick, 2008 ▶); program(s) used to refine structure: *SHELXL97* (Sheldrick, 2008 ▶); molecular graphics: *ORTEP-3* (Farrugia, 1997 ▶); software used to prepare material for publication: *SHELXL97* and *PLATON* (Spek, 2009 ▶).

## Supplementary Material

Crystal structure: contains datablocks global, I. DOI: 10.1107/S1600536809012392/bt2890sup1.cif

Structure factors: contains datablocks I. DOI: 10.1107/S1600536809012392/bt2890Isup2.hkl

Additional supplementary materials:  crystallographic information; 3D view; checkCIF report

## Figures and Tables

**Table 1:** Hydrogen-bond geometry (Å, °)

*D*—H⋯*A*	*D*—H	H⋯*A*	*D*⋯*A*	*D*—H⋯*A*
C9—H9⋯N1	0.93	2.55	3.052 (3)	114
C9′—H9′⋯N1′	0.93	2.50	2.993 (3)	113
C15—H15⋯N17	0.93	2.52	3.280 (4)	139
C15′—H15′⋯N17′	0.93	2.61	3.334 (3)	135
C5—H5⋯Cl2′^i^	0.93	2.81	3.727 (3)	169
N14—H14⋯N17′^ii^	0.73 (3)	2.36 (3)	3.075 (3)	166 (3)
N14′—H14′⋯N17^iii^	0.86 (3)	2.33 (3)	3.157 (3)	160 (3)
C15′—H15′⋯*Cg*5	0.93	3.13	3.798 (3)	131
C23—H23⋯*Cg*8	0.93	2.76	3.620 (3)	155
C23′—H23′⋯*Cg*7	0.93	2.83	3.633 (3)	145

Symmetry codes: (i) 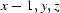; (ii) 
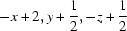
; (iii) 
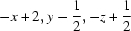
. *Cg*5, *Cg*7 and *Cg*8 are the centroids of the C24–N29, C8–C13 and C8*A*–C13*A* rings, respectively.

**Fig. 1.**
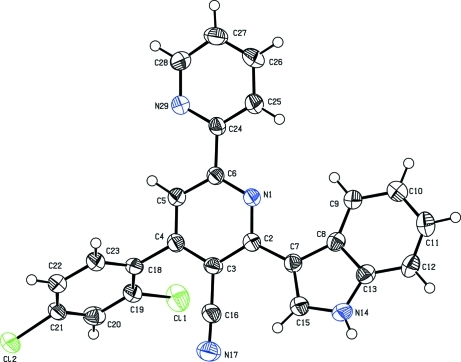
Perspective view of one of the two molecules in the asymmetric unit with displacement ellipsoids drawn at the 50% probability level. The H atoms are shown as small circles of arbitrary radii.

**Fig. 2.**
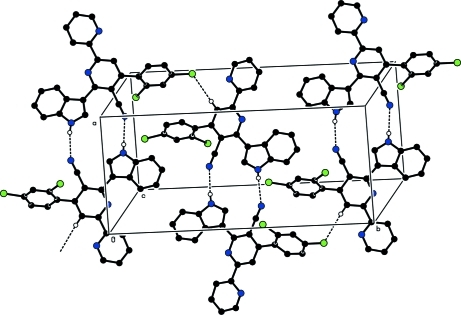
The crystal packing of the molecules viewed down c axis. H atoms not involved in hydrogen bonding have been omitted for clarity.

## supplementary crystallographic information

### Comment

Pyridine derivatives containing multi-functional groups can be used as drugs such as streptonigrin, streptonigrone and lavendamycin which are reported as anticancer drugs, and itavastatin, cerivastatin are reported as the HMG-CoA enzyme inhibitors (Zhou *et al.*, 2008). Indole derivatives are used as bioactive drugs (Stevenson *et al.*, 2000) and they exhibit anti-allergic, central nervous system depressant and muscle relaxant properties (Harris & Uhle 1960; Ho *et al.*, 1986). Indoles have been proved to display high aldose reductase inhibitory activity (Rajeswaran *et al.*, 1999).

The *ORTEP* diagram of the title compound is shown in Fig. 1. In the title compound, there are two crystallographically independent molecules in the asymmetric unit. The two pyridine rings lie in the same plane as can be seen from the dihedral angle of 3.2 (1)° and 8.6 (1)°. The dichlorophenyl and indole rings are twisted away from the bipyridine ring by 64.32 (5)° and 18.46 (4)°, respectively. In the benzene ring of the indole ring system, the endocyclic angels at C12 and C12' are contracted to 117.7 (2)° and 117.9 (3)°, while those at C13 and C13' are expanded to 123.0 (2)° and 122.2 (3)°, respectively. This would appear to be a real effect caused by the fusion of the pyrrole with benzene ring resulting in an angular distortion. The sum of the bond angles around N14(359.3)° and N14'(360.3)° are in accordance with *sp*^2^ hybridization. The bond angles of C3—C16—N17 (178.0 (3))° and C3'-C16'-N17' (178.0 (3))° show the linearity of the cyano group, a feature observed in carbonitrile compounds.

The crystal packing is controlled by C—H···N, C—H···Cl, N—H···N and C—H···π types of intra and intermolecular interactions in addition to van der Waals forces. Atoms N14 and N14' at (*x*, *y*, *z*) donate one proton each to N17 (-*x*, *y* + 1/2, -*z* + 1/2) and N17'(-*x*, *y* - 1/2, -*z* + 1/2) which connects the molecules to form a dimer with a graph-set-motiff *R*^2^_2_(16) (Bernstein *et al.*, 1995). These dimers are linked into a zigzag chain running along *b* axis through intermolecular C5—H5···Cl2' hydrogen bond which is shown in Fig. 2.

### Experimental

A mixture of 4-(2,4-dichlorophenyl)-6-(1*H*-indol-3-yl)-1,4-dihydro -2,2'-bipyridine-5-carbonitrile (1 mmol) and urea oxalate (20 mol%) was irradiated in a microwave oven in ethanol for 5 min. After the completion of the reaction (as monitored by TLC), it was poured into water and extracted with ethyl acetate. The organic layer was dried over sodium sulfate and concentrated under vacuo. The crude product was chromatographed and isolated in 86% yield (90:10, petroleum ether: ethyl acetate). The compound was recrystallized in ethanol.

### Refinement

H atoms bonded to nitrogen were freely refined; those bonded to carbon were positioned geometrically (C—H=0.93 Å) and allowed to ride on their parent atoms, with 1.2*U*_eq_(C).

### Crystal data

**Table:**

C_25_H_14_Cl_2_N_4_	*F*(000) = 1808
*M**_r_* = 441.30	*D*_x_ = 1.452 Mg m^−^^3^
Monoclinic, *P*2_1_/*c*	Mo *K*α radiation, λ = 0.71073 Å
Hall symbol: -P 2ybc	Cell parameters from 4532 reflections
*a* = 10.0307 (12) Å	θ = 1.5–26.0°
*b* = 22.446 (3) Å	µ = 0.34 mm^−^^1^
*c* = 17.932 (3) Å	*T* = 293 K
β = 90.991 (4)°	Block, colourless
*V* = 4036.7 (10) Å^3^	0.30 × 0.25 × 0.20 mm
*Z* = 8	

### Data collection

**Table:**

Bruker Kappa APEXII area-detector diffractometer	7874 independent reflections
Radiation source: fine-focus sealed tube	5586 reflections with *I* > 2σ(*I*)
graphite	*R*_int_ = 0.038
ω and φ scans	θ_max_ = 26.0°, θ_min_ = 1.5°
Absorption correction: multi-scan (*SADABS*; Sheldrick, 2001)	*h* = −12→12
*T*_min_ = 0.902, *T*_max_ = 0.934	*k* = −27→27
38628 measured reflections	*l* = −22→22

### Refinement

**Table:**

Refinement on *F*^2^	Primary atom site location: structure-invariant direct methods
Least-squares matrix: full	Secondary atom site location: difference Fourier map
*R*[*F*^2^ > 2σ(*F*^2^)] = 0.043	Hydrogen site location: inferred from neighbouring sites
*wR*(*F*^2^) = 0.141	H atoms treated by a mixture of independent and constrained refinement
*S* = 1.06	*w* = 1/[σ^2^(*F*_o_^2^) + (0.0749*P*)^2^ + 1.173*P*] where *P* = (*F*_o_^2^ + 2*F*_c_^2^)/3
7874 reflections	(Δ/σ)_max_ = 0.002
567 parameters	Δρ_max_ = 0.52 e Å^−^^3^
0 restraints	Δρ_min_ = −0.41 e Å^−^^3^

### Special details

**Table:**

Geometry. All e.s.d.'s (except the e.s.d. in the dihedral angle between two l.s. planes) are estimated using the full covariance matrix. The cell e.s.d.'s are taken into account individually in the estimation of e.s.d.'s in distances, angles and torsion angles; correlations between e.s.d.'s in cell parameters are only used when they are defined by crystal symmetry. An approximate (isotropic) treatment of cell e.s.d.'s is used for estimating e.s.d.'s involving l.s. planes.
Refinement. Refinement of *F*^2^ against ALL reflections. The weighted *R*-factor *wR* and goodness of fit *S* are based on *F*^2^, conventional *R*-factors *R* are based on *F*, with *F* set to zero for negative *F*^2^. The threshold expression of *F*^2^ > σ(*F*^2^) is used only for calculating *R*-factors(gt) *etc*. and is not relevant to the choice of reflections for refinement. *R*-factors based on *F*^2^ are statistically about twice as large as those based on *F*, and *R*- factors based on ALL data will be even larger.

### Fractional atomic coordinates and isotropic or equivalent isotropic displacement parameters (Å^2^)

**Table:**

	*x*	*y*	*z*	*U*_iso_*/*U*_eq_	
Cl1	0.45103 (8)	0.83228 (3)	−0.01221 (4)	0.0625 (2)	
Cl1'	1.02167 (6)	0.57914 (3)	0.01576 (4)	0.04880 (18)	
Cl2	0.36009 (7)	0.60707 (3)	0.06685 (5)	0.0571 (2)	
Cl2'	0.83340 (8)	0.80040 (3)	0.03850 (6)	0.0715 (3)	
N1	0.20249 (18)	0.98708 (8)	0.13679 (11)	0.0348 (4)	
N1'	0.74187 (17)	0.42415 (8)	0.15496 (11)	0.0344 (4)	
C2	0.3239 (2)	0.96455 (10)	0.15411 (12)	0.0341 (5)	
C2'	0.8626 (2)	0.44706 (10)	0.17186 (12)	0.0324 (5)	
C3	0.3509 (2)	0.90381 (10)	0.14152 (13)	0.0356 (5)	
C3'	0.8898 (2)	0.50742 (10)	0.15868 (13)	0.0344 (5)	
C4	0.2523 (2)	0.86696 (10)	0.11020 (13)	0.0346 (5)	
C4'	0.7929 (2)	0.54340 (10)	0.12346 (13)	0.0331 (5)	
C5	0.1296 (2)	0.89156 (10)	0.09365 (14)	0.0379 (5)	
H5	0.0617	0.8682	0.0733	0.046*	
C5'	0.6708 (2)	0.51797 (10)	0.10687 (13)	0.0360 (5)	
H5'	0.6040	0.5404	0.0837	0.043*	
C6	0.1082 (2)	0.95146 (10)	0.10757 (13)	0.0338 (5)	
C6'	0.6482 (2)	0.45913 (10)	0.12483 (13)	0.0336 (5)	
C7	0.4203 (2)	1.00692 (11)	0.18467 (13)	0.0363 (5)	
C7'	0.9576 (2)	0.40339 (10)	0.20169 (13)	0.0341 (5)	
C8	0.4100 (2)	1.07118 (10)	0.18173 (13)	0.0361 (5)	
C8'	0.9477 (2)	0.33985 (11)	0.19115 (14)	0.0379 (5)	
C9	0.3196 (2)	1.11211 (11)	0.15114 (14)	0.0422 (6)	
H9	0.2435	1.0990	0.1258	0.051*	
C9'	0.8629 (3)	0.30236 (12)	0.15088 (17)	0.0496 (7)	
H9'	0.7916	0.3179	0.1233	0.060*	
C10	0.3444 (3)	1.17177 (12)	0.15879 (16)	0.0486 (6)	
H10	0.2842	1.1990	0.1383	0.058*	
C10'	0.8856 (3)	0.24218 (13)	0.1523 (2)	0.0661 (9)	
H10'	0.8289	0.2171	0.1253	0.079*	
C11	0.4577 (3)	1.19271 (12)	0.19653 (16)	0.0526 (7)	
H11	0.4711	1.2335	0.2016	0.063*	
C11'	0.9914 (3)	0.21783 (13)	0.1930 (2)	0.0681 (9)	
H11'	1.0034	0.1767	0.1937	0.082*	
C12	0.5494 (3)	1.15379 (12)	0.22616 (15)	0.0489 (7)	
H12	0.6263	1.1674	0.2504	0.059*	
C12'	1.0781 (3)	0.25361 (13)	0.23219 (18)	0.0573 (8)	
H12'	1.1499	0.2375	0.2588	0.069*	
C13	0.5240 (2)	1.09366 (11)	0.21879 (14)	0.0399 (6)	
C13'	1.0555 (2)	0.31442 (12)	0.23096 (14)	0.0422 (6)	
N14	0.5971 (2)	1.04600 (10)	0.24325 (13)	0.0462 (6)	
H14	0.662 (3)	1.0477 (14)	0.2631 (17)	0.057 (10)*	
N14'	1.1256 (2)	0.35976 (10)	0.26389 (12)	0.0447 (5)	
H14'	1.197 (3)	0.3551 (13)	0.2910 (16)	0.056 (9)*	
C15	0.5373 (2)	0.99500 (12)	0.22312 (14)	0.0432 (6)	
H15	0.5699	0.9571	0.2336	0.052*	
C15'	1.0688 (2)	0.41225 (12)	0.24621 (13)	0.0398 (6)	
H15'	1.1002	0.4493	0.2619	0.048*	
C16	0.4777 (3)	0.87749 (11)	0.15980 (15)	0.0437 (6)	
C16'	1.0143 (2)	0.53357 (11)	0.18177 (14)	0.0405 (6)	
N17	0.5778 (2)	0.85535 (11)	0.17464 (15)	0.0609 (7)	
N17'	1.1115 (2)	0.55515 (11)	0.20191 (14)	0.0582 (6)	
C18	0.2769 (2)	0.80267 (10)	0.09598 (13)	0.0352 (5)	
C18'	0.8131 (2)	0.60717 (10)	0.10453 (13)	0.0345 (5)	
C19	0.3670 (2)	0.78254 (11)	0.04386 (14)	0.0385 (5)	
C19'	0.9112 (2)	0.62789 (10)	0.05739 (14)	0.0374 (5)	
C20	0.3917 (2)	0.72262 (11)	0.03353 (15)	0.0443 (6)	
H20	0.4535	0.7100	−0.0012	0.053*	
C20'	0.9179 (2)	0.68721 (11)	0.03654 (15)	0.0431 (6)	
H20'	0.9843	0.7005	0.0050	0.052*	
C21	0.3230 (2)	0.68196 (11)	0.07557 (14)	0.0408 (6)	
C21'	0.8242 (3)	0.72612 (11)	0.06352 (16)	0.0452 (6)	
C22	0.2279 (2)	0.70021 (11)	0.12538 (15)	0.0435 (6)	
H22	0.1788	0.6723	0.1517	0.052*	
C22'	0.7246 (3)	0.70747 (11)	0.11006 (16)	0.0491 (7)	
H22'	0.6617	0.7343	0.1274	0.059*	
C23	0.2068 (2)	0.76007 (11)	0.13554 (14)	0.0407 (6)	
H23	0.1439	0.7724	0.1698	0.049*	
C23'	0.7198 (3)	0.64864 (11)	0.13038 (15)	0.0447 (6)	
H23'	0.6531	0.6359	0.1621	0.054*	
C24	−0.0246 (2)	0.97866 (10)	0.09155 (13)	0.0354 (5)	
C24'	0.5145 (2)	0.43164 (10)	0.11345 (13)	0.0326 (5)	
C25	−0.0512 (3)	1.03740 (11)	0.10704 (16)	0.0472 (6)	
H25	0.0152	1.0620	0.1267	0.057*	
C25'	0.4913 (2)	0.37279 (11)	0.13089 (15)	0.0437 (6)	
H25'	0.5606	0.3483	0.1472	0.052*	
C26	−0.1781 (3)	1.05944 (13)	0.09294 (17)	0.0558 (7)	
H26	−0.1982	1.0990	0.1031	0.067*	
C26'	0.3625 (3)	0.35088 (12)	0.12371 (16)	0.0500 (7)	
H26'	0.3439	0.3114	0.1352	0.060*	
C27	−0.2730 (3)	1.02249 (13)	0.06407 (17)	0.0531 (7)	
H27	−0.3592	1.0361	0.0544	0.064*	
C27'	0.2636 (2)	0.38809 (12)	0.09957 (14)	0.0432 (6)	
H27'	0.1761	0.3747	0.0950	0.052*	
C28	−0.2384 (3)	0.96470 (13)	0.04952 (17)	0.0515 (7)	
H28	−0.3034	0.9396	0.0294	0.062*	
C28'	0.2955 (2)	0.44533 (12)	0.08230 (15)	0.0438 (6)	
H28'	0.2275	0.4702	0.0651	0.053*	
N29	−0.1168 (2)	0.94216 (9)	0.06248 (13)	0.0453 (5)	
N29'	0.41791 (19)	0.46807 (9)	0.08843 (12)	0.0402 (5)	

### Atomic displacement parameters (Å^2^)

**Table:**

	*U*^11^	*U*^22^	*U*^33^	*U*^12^	*U*^13^	*U*^23^
Cl1	0.0732 (5)	0.0578 (4)	0.0573 (4)	−0.0020 (4)	0.0262 (4)	0.0068 (3)
Cl1'	0.0403 (3)	0.0521 (4)	0.0542 (4)	0.0078 (3)	0.0067 (3)	0.0029 (3)
Cl2	0.0517 (4)	0.0350 (3)	0.0842 (5)	0.0091 (3)	−0.0060 (4)	−0.0091 (3)
Cl2'	0.0753 (5)	0.0338 (4)	0.1049 (7)	−0.0035 (3)	−0.0140 (5)	0.0104 (4)
N1	0.0330 (10)	0.0340 (10)	0.0375 (11)	0.0009 (8)	0.0003 (8)	−0.0003 (9)
N1'	0.0271 (9)	0.0352 (10)	0.0408 (11)	0.0017 (8)	−0.0008 (8)	0.0008 (9)
C2	0.0345 (12)	0.0363 (12)	0.0316 (12)	0.0002 (10)	0.0013 (10)	0.0017 (10)
C2'	0.0293 (11)	0.0369 (12)	0.0310 (12)	0.0018 (9)	0.0015 (9)	−0.0005 (10)
C3	0.0305 (11)	0.0380 (13)	0.0383 (13)	0.0029 (10)	−0.0001 (10)	−0.0001 (10)
C3'	0.0290 (11)	0.0392 (13)	0.0350 (12)	−0.0016 (10)	−0.0013 (9)	−0.0003 (10)
C4	0.0341 (12)	0.0345 (12)	0.0355 (13)	0.0012 (10)	0.0047 (10)	−0.0001 (10)
C4'	0.0312 (11)	0.0330 (12)	0.0351 (13)	−0.0007 (9)	−0.0012 (9)	−0.0011 (10)
C5	0.0332 (12)	0.0358 (13)	0.0448 (14)	−0.0016 (10)	0.0013 (10)	−0.0013 (11)
C5'	0.0305 (12)	0.0358 (13)	0.0417 (13)	0.0026 (10)	−0.0035 (10)	0.0043 (10)
C6	0.0314 (12)	0.0339 (12)	0.0360 (13)	0.0004 (10)	0.0041 (10)	0.0019 (10)
C6'	0.0292 (11)	0.0362 (12)	0.0352 (13)	0.0018 (9)	−0.0017 (9)	−0.0004 (10)
C7	0.0361 (12)	0.0386 (13)	0.0340 (13)	0.0002 (10)	0.0001 (10)	−0.0013 (10)
C7'	0.0284 (11)	0.0390 (13)	0.0349 (12)	0.0035 (9)	0.0008 (9)	0.0051 (10)
C8	0.0334 (12)	0.0406 (13)	0.0343 (13)	−0.0016 (10)	0.0017 (10)	−0.0035 (10)
C8'	0.0298 (12)	0.0420 (13)	0.0421 (14)	0.0070 (10)	0.0031 (10)	0.0060 (11)
C9	0.0354 (13)	0.0425 (14)	0.0486 (15)	0.0003 (11)	−0.0015 (11)	0.0004 (12)
C9'	0.0389 (14)	0.0434 (15)	0.0662 (19)	0.0045 (11)	−0.0047 (13)	−0.0024 (13)
C10	0.0467 (15)	0.0417 (14)	0.0573 (17)	0.0029 (12)	0.0011 (13)	0.0037 (12)
C10'	0.0569 (18)	0.0440 (16)	0.097 (3)	0.0054 (14)	−0.0042 (17)	−0.0104 (16)
C11	0.0599 (17)	0.0383 (14)	0.0596 (18)	−0.0090 (13)	0.0019 (14)	−0.0017 (13)
C11'	0.069 (2)	0.0391 (16)	0.097 (3)	0.0152 (15)	0.0030 (19)	0.0016 (16)
C12	0.0498 (15)	0.0476 (15)	0.0492 (16)	−0.0113 (12)	−0.0048 (12)	−0.0068 (13)
C12'	0.0514 (17)	0.0542 (17)	0.0664 (19)	0.0215 (14)	0.0034 (15)	0.0135 (15)
C13	0.0379 (13)	0.0435 (14)	0.0381 (13)	−0.0022 (11)	−0.0012 (11)	−0.0036 (11)
C13'	0.0351 (13)	0.0477 (14)	0.0439 (14)	0.0076 (11)	0.0033 (11)	0.0103 (12)
N14	0.0397 (13)	0.0494 (14)	0.0490 (14)	−0.0009 (11)	−0.0137 (11)	−0.0028 (10)
N14'	0.0338 (11)	0.0575 (14)	0.0426 (12)	0.0090 (10)	−0.0059 (10)	0.0091 (11)
C15	0.0415 (14)	0.0438 (14)	0.0442 (15)	0.0032 (11)	−0.0062 (11)	−0.0001 (12)
C15'	0.0372 (13)	0.0465 (14)	0.0357 (13)	0.0044 (11)	−0.0013 (10)	0.0029 (11)
C16	0.0412 (14)	0.0415 (14)	0.0483 (15)	0.0028 (11)	−0.0054 (12)	−0.0072 (12)
C16'	0.0385 (13)	0.0399 (13)	0.0429 (14)	−0.0022 (11)	−0.0076 (11)	0.0062 (11)
N17	0.0496 (14)	0.0586 (15)	0.0739 (18)	0.0139 (12)	−0.0148 (12)	−0.0155 (13)
N17'	0.0469 (14)	0.0605 (15)	0.0666 (16)	−0.0129 (12)	−0.0207 (12)	0.0127 (13)
C18	0.0320 (12)	0.0350 (12)	0.0385 (13)	0.0039 (10)	−0.0013 (10)	−0.0007 (10)
C18'	0.0316 (12)	0.0335 (12)	0.0382 (13)	−0.0033 (10)	−0.0077 (10)	−0.0022 (10)
C19	0.0385 (13)	0.0389 (13)	0.0381 (13)	0.0023 (10)	0.0030 (10)	−0.0001 (11)
C19'	0.0332 (12)	0.0374 (13)	0.0413 (14)	−0.0004 (10)	−0.0071 (10)	−0.0016 (11)
C20	0.0407 (14)	0.0474 (15)	0.0448 (15)	0.0079 (12)	0.0024 (11)	−0.0085 (12)
C20'	0.0394 (13)	0.0422 (14)	0.0474 (15)	−0.0088 (11)	−0.0064 (11)	0.0039 (12)
C21	0.0384 (13)	0.0340 (13)	0.0498 (15)	0.0078 (10)	−0.0104 (11)	−0.0044 (11)
C21'	0.0493 (15)	0.0301 (12)	0.0557 (17)	−0.0032 (11)	−0.0143 (13)	−0.0011 (11)
C22	0.0396 (13)	0.0370 (13)	0.0540 (16)	0.0010 (11)	0.0004 (12)	0.0047 (12)
C22'	0.0453 (15)	0.0378 (14)	0.0639 (18)	0.0051 (12)	−0.0039 (13)	−0.0102 (13)
C23	0.0341 (12)	0.0412 (13)	0.0471 (15)	0.0029 (10)	0.0074 (11)	−0.0008 (11)
C23'	0.0420 (14)	0.0379 (14)	0.0540 (16)	−0.0009 (11)	−0.0022 (12)	−0.0042 (12)
C24	0.0346 (12)	0.0356 (12)	0.0361 (13)	0.0006 (10)	0.0027 (10)	0.0042 (10)
C24'	0.0297 (11)	0.0322 (12)	0.0359 (12)	0.0004 (9)	0.0004 (9)	−0.0017 (10)
C25	0.0441 (14)	0.0373 (14)	0.0602 (17)	0.0026 (11)	0.0002 (12)	−0.0029 (12)
C25'	0.0341 (12)	0.0362 (13)	0.0609 (17)	0.0012 (10)	−0.0027 (11)	0.0045 (12)
C26	0.0502 (16)	0.0444 (15)	0.073 (2)	0.0140 (13)	0.0011 (14)	−0.0004 (14)
C26'	0.0437 (15)	0.0406 (14)	0.0658 (18)	−0.0089 (12)	0.0011 (13)	0.0059 (13)
C27	0.0385 (14)	0.0565 (17)	0.0643 (19)	0.0120 (13)	0.0006 (13)	0.0111 (14)
C27'	0.0311 (12)	0.0534 (16)	0.0449 (15)	−0.0092 (11)	−0.0020 (11)	−0.0012 (12)
C28	0.0352 (14)	0.0536 (16)	0.0655 (19)	−0.0027 (12)	−0.0071 (12)	0.0070 (14)
C28'	0.0304 (12)	0.0502 (15)	0.0506 (15)	0.0023 (11)	−0.0074 (11)	0.0024 (12)
N29	0.0360 (11)	0.0408 (12)	0.0590 (14)	0.0006 (9)	−0.0040 (10)	0.0030 (10)
N29'	0.0314 (10)	0.0377 (11)	0.0512 (13)	0.0009 (9)	−0.0079 (9)	0.0027 (9)

### Geometric parameters (Å, °)

**Table:**

Cl1—C19	1.731 (3)	C12'—H12'	0.9300
Cl1'—C19'	1.735 (2)	C13—N14	1.365 (3)
Cl2—C21	1.729 (2)	C13'—N14'	1.365 (3)
Cl2'—C21'	1.729 (3)	N14—C15	1.339 (3)
N1—C6	1.338 (3)	N14—H14	0.73 (3)
N1—C2	1.350 (3)	N14'—C15'	1.344 (3)
N1'—C6'	1.332 (3)	N14'—H14'	0.86 (3)
N1'—C2'	1.346 (3)	C15—H15	0.9300
C2—C3	1.409 (3)	C15'—H15'	0.9300
C2—C7	1.457 (3)	C16—N17	1.147 (3)
C2'—C3'	1.403 (3)	C16'—N17'	1.142 (3)
C2'—C7'	1.462 (3)	C18—C19	1.387 (3)
C3—C4	1.400 (3)	C18—C23	1.389 (3)
C3—C16	1.435 (3)	C18'—C19'	1.389 (3)
C3'—C4'	1.406 (3)	C18'—C23'	1.404 (3)
C3'—C16'	1.434 (3)	C19—C20	1.380 (3)
C4—C5	1.377 (3)	C19'—C20'	1.385 (3)
C4—C18	1.487 (3)	C20—C21	1.376 (4)
C4'—C5'	1.379 (3)	C20—H20	0.9300
C4'—C18'	1.486 (3)	C20'—C21'	1.377 (4)
C5—C6	1.385 (3)	C20'—H20'	0.9300
C5—H5	0.9300	C21—C22	1.380 (4)
C5'—C6'	1.379 (3)	C21'—C22'	1.378 (4)
C5'—H5'	0.9300	C22—C23	1.373 (3)
C6—C24	1.489 (3)	C22—H22	0.9300
C6'—C24'	1.487 (3)	C22'—C23'	1.371 (4)
C7—C15	1.376 (3)	C22'—H22'	0.9300
C7—C8	1.447 (3)	C23—H23	0.9300
C7'—C15'	1.375 (3)	C23'—H23'	0.9300
C7'—C8'	1.442 (3)	C24—N29	1.335 (3)
C8—C9	1.396 (3)	C24—C25	1.374 (3)
C8—C13	1.406 (3)	C24'—N29'	1.339 (3)
C8'—C9'	1.390 (4)	C24'—C25'	1.378 (3)
C8'—C13'	1.406 (3)	C25—C26	1.386 (4)
C9—C10	1.368 (4)	C25—H25	0.9300
C9—H9	0.9300	C25'—C26'	1.386 (3)
C9'—C10'	1.370 (4)	C25'—H25'	0.9300
C9'—H9'	0.9300	C26—C27	1.358 (4)
C10—C11	1.394 (4)	C26—H26	0.9300
C10—H10	0.9300	C26'—C27'	1.361 (4)
C10'—C11'	1.390 (4)	C26'—H26'	0.9300
C10'—H10'	0.9300	C27—C28	1.369 (4)
C11—C12	1.370 (4)	C27—H27	0.9300
C11—H11	0.9300	C27'—C28'	1.361 (4)
C11'—C12'	1.369 (4)	C27'—H27'	0.9300
C11'—H11'	0.9300	C28—N29	1.337 (3)
C12—C13	1.380 (4)	C28—H28	0.9300
C12—H12	0.9300	C28'—N29'	1.332 (3)
C12'—C13'	1.384 (4)	C28'—H28'	0.9300
			
C6—N1—C2	119.67 (19)	C15'—N14'—H14'	126 (2)
C6'—N1'—C2'	119.48 (19)	C13'—N14'—H14'	124.5 (19)
N1—C2—C3	120.1 (2)	N14—C15—C7	110.1 (2)
N1—C2—C7	115.6 (2)	N14—C15—H15	125.0
C3—C2—C7	124.3 (2)	C7—C15—H15	125.0
N1'—C2'—C3'	120.5 (2)	N14'—C15'—C7'	110.3 (2)
N1'—C2'—C7'	113.9 (2)	N14'—C15'—H15'	124.9
C3'—C2'—C7'	125.6 (2)	C7'—C15'—H15'	124.9
C4—C3—C2	120.0 (2)	N17—C16—C3	178.6 (3)
C4—C3—C16	117.8 (2)	N17'—C16'—C3'	178.0 (3)
C2—C3—C16	122.3 (2)	C19—C18—C23	117.5 (2)
C2'—C3'—C4'	119.7 (2)	C19—C18—C4	122.9 (2)
C2'—C3'—C16'	121.2 (2)	C23—C18—C4	119.6 (2)
C4'—C3'—C16'	119.1 (2)	C19'—C18'—C23'	117.5 (2)
C5—C4—C3	118.2 (2)	C19'—C18'—C4'	124.2 (2)
C5—C4—C18	120.2 (2)	C23'—C18'—C4'	118.0 (2)
C3—C4—C18	121.6 (2)	C20—C19—C18	121.9 (2)
C5'—C4'—C3'	117.7 (2)	C20—C19—Cl1	117.41 (19)
C5'—C4'—C18'	118.3 (2)	C18—C19—Cl1	120.71 (19)
C3'—C4'—C18'	124.0 (2)	C20'—C19'—C18'	121.6 (2)
C4—C5—C6	119.4 (2)	C20'—C19'—Cl1'	117.12 (19)
C4—C5—H5	120.3	C18'—C19'—Cl1'	121.02 (18)
C6—C5—H5	120.3	C21—C20—C19	118.7 (2)
C4'—C5'—C6'	119.7 (2)	C21—C20—H20	120.7
C4'—C5'—H5'	120.2	C19—C20—H20	120.7
C6'—C5'—H5'	120.2	C21'—C20'—C19'	118.6 (2)
N1—C6—C5	122.7 (2)	C21'—C20'—H20'	120.7
N1—C6—C24	117.0 (2)	C19'—C20'—H20'	120.7
C5—C6—C24	120.3 (2)	C20—C21—C22	121.0 (2)
N1'—C6'—C5'	122.8 (2)	C20—C21—Cl2	119.0 (2)
N1'—C6'—C24'	116.1 (2)	C22—C21—Cl2	119.9 (2)
C5'—C6'—C24'	121.1 (2)	C20'—C21'—C22'	121.8 (2)
C15—C7—C8	105.8 (2)	C20'—C21'—Cl2'	118.7 (2)
C15—C7—C2	128.0 (2)	C22'—C21'—Cl2'	119.5 (2)
C8—C7—C2	126.2 (2)	C23—C22—C21	119.1 (2)
C15'—C7'—C8'	105.8 (2)	C23—C22—H22	120.5
C15'—C7'—C2'	129.2 (2)	C21—C22—H22	120.5
C8'—C7'—C2'	124.9 (2)	C23'—C22'—C21'	118.9 (2)
C9—C8—C13	117.8 (2)	C23'—C22'—H22'	120.6
C9—C8—C7	135.7 (2)	C21'—C22'—H22'	120.6
C13—C8—C7	106.4 (2)	C22—C23—C18	121.7 (2)
C9'—C8'—C13'	118.4 (2)	C22—C23—H23	119.2
C9'—C8'—C7'	135.0 (2)	C18—C23—H23	119.2
C13'—C8'—C7'	106.5 (2)	C22'—C23'—C18'	121.6 (3)
C10—C9—C8	119.3 (2)	C22'—C23'—H23'	119.2
C10—C9—H9	120.3	C18'—C23'—H23'	119.2
C8—C9—H9	120.3	N29—C24—C25	122.1 (2)
C10'—C9'—C8'	119.2 (3)	N29—C24—C6	115.8 (2)
C10'—C9'—H9'	120.4	C25—C24—C6	122.0 (2)
C8'—C9'—H9'	120.4	N29'—C24'—C25'	122.5 (2)
C9—C10—C11	121.6 (3)	N29'—C24'—C6'	115.98 (19)
C9—C10—H10	119.2	C25'—C24'—C6'	121.5 (2)
C11—C10—H10	119.2	C24—C25—C26	119.1 (2)
C9'—C10'—C11'	121.5 (3)	C24—C25—H25	120.4
C9'—C10'—H10'	119.3	C26—C25—H25	120.4
C11'—C10'—H10'	119.3	C24'—C25'—C26'	118.7 (2)
C12—C11—C10	120.7 (2)	C24'—C25'—H25'	120.7
C12—C11—H11	119.7	C26'—C25'—H25'	120.7
C10—C11—H11	119.7	C27—C26—C25	119.2 (3)
C12'—C11'—C10'	120.8 (3)	C27—C26—H26	120.4
C12'—C11'—H11'	119.6	C25—C26—H26	120.4
C10'—C11'—H11'	119.6	C27'—C26'—C25'	119.0 (2)
C11—C12—C13	117.7 (2)	C27'—C26'—H26'	120.5
C11—C12—H12	121.1	C25'—C26'—H26'	120.5
C13—C12—H12	121.1	C26—C27—C28	118.2 (2)
C11'—C12'—C13'	117.9 (3)	C26—C27—H27	120.9
C11'—C12'—H12'	121.0	C28—C27—H27	120.9
C13'—C12'—H12'	121.0	C28'—C27'—C26'	118.6 (2)
N14—C13—C12	129.7 (2)	C28'—C27'—H27'	120.7
N14—C13—C8	107.4 (2)	C26'—C27'—H27'	120.7
C12—C13—C8	123.0 (2)	N29—C28—C27	124.0 (3)
N14'—C13'—C12'	130.2 (2)	N29—C28—H28	118.0
N14'—C13'—C8'	107.6 (2)	C27—C28—H28	118.0
C12'—C13'—C8'	122.2 (3)	N29'—C28'—C27'	124.2 (2)
C15—N14—C13	110.3 (2)	N29'—C28'—H28'	117.9
C15—N14—H14	124 (2)	C27'—C28'—H28'	117.9
C13—N14—H14	125 (2)	C24—N29—C28	117.4 (2)
C15'—N14'—C13'	109.8 (2)	C28'—N29'—C24'	117.0 (2)
			
C6—N1—C2—C3	−0.1 (3)	C8—C13—N14—C15	0.8 (3)
C6—N1—C2—C7	179.2 (2)	C12'—C13'—N14'—C15'	178.6 (3)
C6'—N1'—C2'—C3'	0.6 (3)	C8'—C13'—N14'—C15'	−0.8 (3)
C6'—N1'—C2'—C7'	−177.6 (2)	C13—N14—C15—C7	−0.5 (3)
N1—C2—C3—C4	0.8 (3)	C8—C7—C15—N14	0.0 (3)
C7—C2—C3—C4	−178.5 (2)	C2—C7—C15—N14	−178.8 (2)
N1—C2—C3—C16	−179.2 (2)	C13'—N14'—C15'—C7'	0.9 (3)
C7—C2—C3—C16	1.6 (4)	C8'—C7'—C15'—N14'	−0.6 (3)
N1'—C2'—C3'—C4'	−3.8 (3)	C2'—C7'—C15'—N14'	177.3 (2)
C7'—C2'—C3'—C4'	174.2 (2)	C4—C3—C16—N17	−28 (12)
N1'—C2'—C3'—C16'	175.0 (2)	C2—C3—C16—N17	152 (12)
C7'—C2'—C3'—C16'	−7.0 (4)	C2'—C3'—C16'—N17'	−103 (8)
C2—C3—C4—C5	−1.1 (3)	C4'—C3'—C16'—N17'	76 (8)
C16—C3—C4—C5	178.9 (2)	C5—C4—C18—C19	114.7 (3)
C2—C3—C4—C18	180.0 (2)	C3—C4—C18—C19	−66.4 (3)
C16—C3—C4—C18	−0.1 (3)	C5—C4—C18—C23	−64.7 (3)
C2'—C3'—C4'—C5'	3.5 (3)	C3—C4—C18—C23	114.2 (3)
C16'—C3'—C4'—C5'	−175.4 (2)	C5'—C4'—C18'—C19'	−121.2 (3)
C2'—C3'—C4'—C18'	−177.8 (2)	C3'—C4'—C18'—C19'	60.1 (3)
C16'—C3'—C4'—C18'	3.4 (4)	C5'—C4'—C18'—C23'	52.8 (3)
C3—C4—C5—C6	0.7 (3)	C3'—C4'—C18'—C23'	−126.0 (3)
C18—C4—C5—C6	179.7 (2)	C23—C18—C19—C20	−3.0 (4)
C3'—C4'—C5'—C6'	−0.2 (3)	C4—C18—C19—C20	177.6 (2)
C18'—C4'—C5'—C6'	−179.0 (2)	C23—C18—C19—Cl1	175.85 (18)
C2—N1—C6—C5	−0.3 (3)	C4—C18—C19—Cl1	−3.6 (3)
C2—N1—C6—C24	178.4 (2)	C23'—C18'—C19'—C20'	0.2 (3)
C4—C5—C6—N1	0.0 (4)	C4'—C18'—C19'—C20'	174.2 (2)
C4—C5—C6—C24	−178.7 (2)	C23'—C18'—C19'—Cl1'	−174.10 (18)
C2'—N1'—C6'—C5'	2.9 (3)	C4'—C18'—C19'—Cl1'	−0.1 (3)
C2'—N1'—C6'—C24'	−175.3 (2)	C18—C19—C20—C21	1.0 (4)
C4'—C5'—C6'—N1'	−3.1 (4)	Cl1—C19—C20—C21	−177.85 (19)
C4'—C5'—C6'—C24'	175.0 (2)	C18'—C19'—C20'—C21'	−0.1 (4)
N1—C2—C7—C15	163.0 (2)	Cl1'—C19'—C20'—C21'	174.42 (19)
C3—C2—C7—C15	−17.8 (4)	C19—C20—C21—C22	2.2 (4)
N1—C2—C7—C8	−15.6 (3)	C19—C20—C21—Cl2	−176.34 (19)
C3—C2—C7—C8	163.7 (2)	C19'—C20'—C21'—C22'	−0.3 (4)
N1'—C2'—C7'—C15'	−156.2 (2)	C19'—C20'—C21'—Cl2'	179.25 (19)
C3'—C2'—C7'—C15'	25.6 (4)	C20—C21—C22—C23	−3.3 (4)
N1'—C2'—C7'—C8'	21.3 (3)	Cl2—C21—C22—C23	175.3 (2)
C3'—C2'—C7'—C8'	−156.9 (2)	C20'—C21'—C22'—C23'	0.6 (4)
C15—C7—C8—C9	179.0 (3)	Cl2'—C21'—C22'—C23'	−179.0 (2)
C2—C7—C8—C9	−2.2 (4)	C21—C22—C23—C18	1.2 (4)
C15—C7—C8—C13	0.5 (3)	C19—C18—C23—C22	1.8 (4)
C2—C7—C8—C13	179.3 (2)	C4—C18—C23—C22	−178.7 (2)
C15'—C7'—C8'—C9'	−177.3 (3)	C21'—C22'—C23'—C18'	−0.5 (4)
C2'—C7'—C8'—C9'	4.7 (4)	C19'—C18'—C23'—C22'	0.1 (4)
C15'—C7'—C8'—C13'	0.1 (3)	C4'—C18'—C23'—C22'	−174.3 (2)
C2'—C7'—C8'—C13'	−177.9 (2)	N1—C6—C24—N29	−180.0 (2)
C13—C8—C9—C10	−0.6 (4)	C5—C6—C24—N29	−1.2 (3)
C7—C8—C9—C10	−179.0 (3)	N1—C6—C24—C25	−1.1 (3)
C13'—C8'—C9'—C10'	1.1 (4)	C5—C6—C24—C25	177.7 (2)
C7'—C8'—C9'—C10'	178.3 (3)	N1'—C6'—C24'—N29'	175.1 (2)
C8—C9—C10—C11	−0.1 (4)	C5'—C6'—C24'—N29'	−3.0 (3)
C8'—C9'—C10'—C11'	0.0 (5)	N1'—C6'—C24'—C25'	−2.1 (3)
C9—C10—C11—C12	1.2 (4)	C5'—C6'—C24'—C25'	179.7 (2)
C9'—C10'—C11'—C12'	−1.2 (5)	N29—C24—C25—C26	0.8 (4)
C10—C11—C12—C13	−1.5 (4)	C6—C24—C25—C26	−178.0 (2)
C10'—C11'—C12'—C13'	1.2 (5)	N29'—C24'—C25'—C26'	−1.4 (4)
C11—C12—C13—N14	−179.4 (3)	C6'—C24'—C25'—C26'	175.7 (2)
C11—C12—C13—C8	0.8 (4)	C24—C25—C26—C27	−0.1 (4)
C9—C8—C13—N14	−179.6 (2)	C24'—C25'—C26'—C27'	0.1 (4)
C7—C8—C13—N14	−0.8 (3)	C25—C26—C27—C28	−0.5 (4)
C9—C8—C13—C12	0.2 (4)	C25'—C26'—C27'—C28'	1.0 (4)
C7—C8—C13—C12	179.0 (2)	C26—C27—C28—N29	0.5 (5)
C11'—C12'—C13'—N14'	−179.3 (3)	C26'—C27'—C28'—N29'	−1.0 (4)
C11'—C12'—C13'—C8'	0.0 (4)	C25—C24—N29—C28	−0.8 (4)
C9'—C8'—C13'—N14'	178.4 (2)	C6—C24—N29—C28	178.0 (2)
C7'—C8'—C13'—N14'	0.4 (3)	C27—C28—N29—C24	0.2 (4)
C9'—C8'—C13'—C12'	−1.1 (4)	C27'—C28'—N29'—C24'	−0.2 (4)
C7'—C8'—C13'—C12'	−179.1 (2)	C25'—C24'—N29'—C28'	1.4 (4)
C12—C13—N14—C15	−179.0 (3)	C6'—C24'—N29'—C28'	−175.9 (2)

### Hydrogen-bond geometry (Å, °)

**Table:**

*D*—H···*A*	*D*—H	H···*A*	*D*···*A*	*D*—H···*A*
C9—H9···N1	0.93	2.55	3.052 (3)	114
C9'—H9'···N1'	0.93	2.50	2.993 (3)	113
C15—H15···N17	0.93	2.52	3.280 (4)	139
C15'—H15'···N17'	0.93	2.61	3.334 (3)	135
C5—H5···Cl2'^i^	0.93	2.81	3.727 (3)	169
N14—H14···N17'^ii^	0.73 (3)	2.36 (3)	3.075 (3)	166 (3)
N14'—H14'···N17^iii^	0.86 (3)	2.33 (3)	3.157 (3)	160 (3)
C15'—H15'···Cg5	0.93	3.13	3.798 (3)	131
C23—H23···Cg8	0.93	2.76	3.620 (3)	155
C23'—H23'···Cg7	0.93	2.83	3.633 (3)	145

Symmetry codes:  (i) *x*−1, *y*, *z*; (ii) −*x*+2, *y*+1/2, −*z*+1/2; (iii) −*x*+2, *y*−1/2, −*z*+1/2.
